# The Approach to Patients with Disorders of Sex Development (DSD) in the Era of Precision Medicine: The Careful Use of Terminology

**DOI:** 10.17925/EE.2024.20.2.4

**Published:** 2024-03-28

**Authors:** Rodolfo A Rey

**Affiliations:** Centro de Investigaciones Endocrinológicas “Dr. César Bergadá” (CEDIE), CONICET – FEI – División de Endocrinología, Hospital de Niños Ricardo Gutiérrez, Buenos Aires, Argentina

**Keywords:** Ambiguous genitalia, atypical genitalia, differences of sex development, disorders of sex development (DSD), personalized medicine, precision medicine

## Abstract

The term “DSD” was coined for “disorders of sex development”, referring to conditions where the chromosomal, gonadal and/or genital sex is discordant or ambiguous, to replace terms considered imprecise and stigmatizing. Recently, the term “disorder” has been questioned and the term “differences” has been proposed as not stigmatizing, reflecting that the term DSD should be depathologized. In this opinion article, I discuss the importance of using precise technical terminologies amongst healthcare professionals, in the era of “precision medicine”, to avoid misleading diagnoses or classifications while being extremely careful to use sensitive terminologies when interacting with patients and their families. On the other hand, I challenge the concept that DSD are not disorders.

The pioneering work carried out by the French scientist Alfred Jost in the mid-20th century clearly showed that, in the mammalian foetus, the internal and external genitalia virilize whenever the undifferentiated gonads develop into testes, whereas they undergo the female pathway when no testicular tissue is present, regardless of the existence or absence of the ovarian tissue (*Figure 1A–C*).^1,2^ In fact, the foetal testis secretes two discrete hormones, testosterone and anti-Müllerian hormone (AMH), which are responsible for genital virilization. This knowledge sets the basis for the understanding of conditions in mammalian species, for instance, freemartinism, and particularly in humans in whom the gonads and/or the genitalia do not follow the expected complete differentiation.^3^ These conditions, known as “intersex” for the whole second half of the 20th century and renamed as “disorders of sex development” (DSD) by the International Consensus Conference on Intersex held in Chicago, IL, USA in 2005, are characterized by a discordance between chromosomal, gonadal and internal and/or external genital sex.^4,5^ To explain it briefly, in the vast majority of mammals, including humans, when the chromosomal sex is 46,XY, the testes differentiate and secrete AMH, which leads to the regression of Müllerian ducts, and testosterone, which is responsible for the differentiation of Wolffian ducts into the epididymides, *vasa deferentia* and seminal vesicles and the virilization of the urogenital sinus and the primordia of the external genitalia (i.e. the genital tubercle, the urethral folds and the labioscrotal folds; *Figure 1B*).^6^ On the other hand, when the chromosomal sex is 46,XX, the gonadal anlagen differentiate into ovaries, which do not secrete relevant amounts of gonadal hormones; therefore, Müllerian ducts give rise to the fallopian tubes, the uterus and the upper part of the vagina; the urogenital sinus gives rise to the lower part of the vagina and the primordia of the external genitalia undergo the female pathway (*Figure 1C*). In approximately 1 in 5,000 individuals, the process of gonadal and/or genital differentiation occurs differently leading to “intersex” conditions or DSD (*Figure 1C–G*).^7^

The most frequent single cause of discordance between the chromosomal/gonadal sex and genital sex is congenital adrenal hyperplasia, occurring in 46,XX individuals. The condition is most frequently due to a defect in adrenal steroidogenesis, leading to an overproduction of androgens that virilize the external genitalia despite the existence of two ovaries (*Figure 1G*).^8^ In persons with a 46,XY karyotype, the lack of virilization may result from a disorder of testis differentiation – known as gonadal dysgenesis (*Figure 1C,D*) – or from specific defects in gonadal hormone synthesis or action (*Figure 1E,F*). Finally, atypical karyotypes, such as 46,XX/46,XY, 45,X/46,XY or other sex chromosome mosaicisms, may be associated with different forms of gonadal dysgenesis, including ovotesticular differentiation (i.e. the existence of both ovarian and testicular tissues in the same individual; *Figure 1D*).^6^ Therefore, according to the Chicago consensus, DSD have been classified into 46,XX DSD, 46,XY DSD and chromosomal DSD.

One of the goals of the Chicago consensus was to replace terms considered imprecise and stigmatizing, such as intersex, hermaphroditism, pseudohermaphroditism and sex reversal. It was agreed that a more appropriate terminology should be (1) precise when applying definitions and diagnostic labels, (2) flexible to incorporate new information, yet robust enough to maintain a consistent framework, (3) descriptive and reflecting genetic aetiology, (4) valued by clinicians and scientists, (5) understandable to individuals and their families and (6) sensitive to the concerns of individuals with these conditions.^4,5^ The broad term DSD has been generally accepted by healthcare professionals, although not universally by patient support groups, some of whom consider that DSD are not medical conditions and should therefore be “depathologized” and called “differences of sex development”.^9^ However, the largest evidence-based study performed and published to date, which evaluated patient-reported outcomes in 1,040 individuals with DSD, found that approximately 70% of the participants thought that the term “disorders of sex development” applied to their condition or that they felt neutral about it.^10^

**Figure 1:**
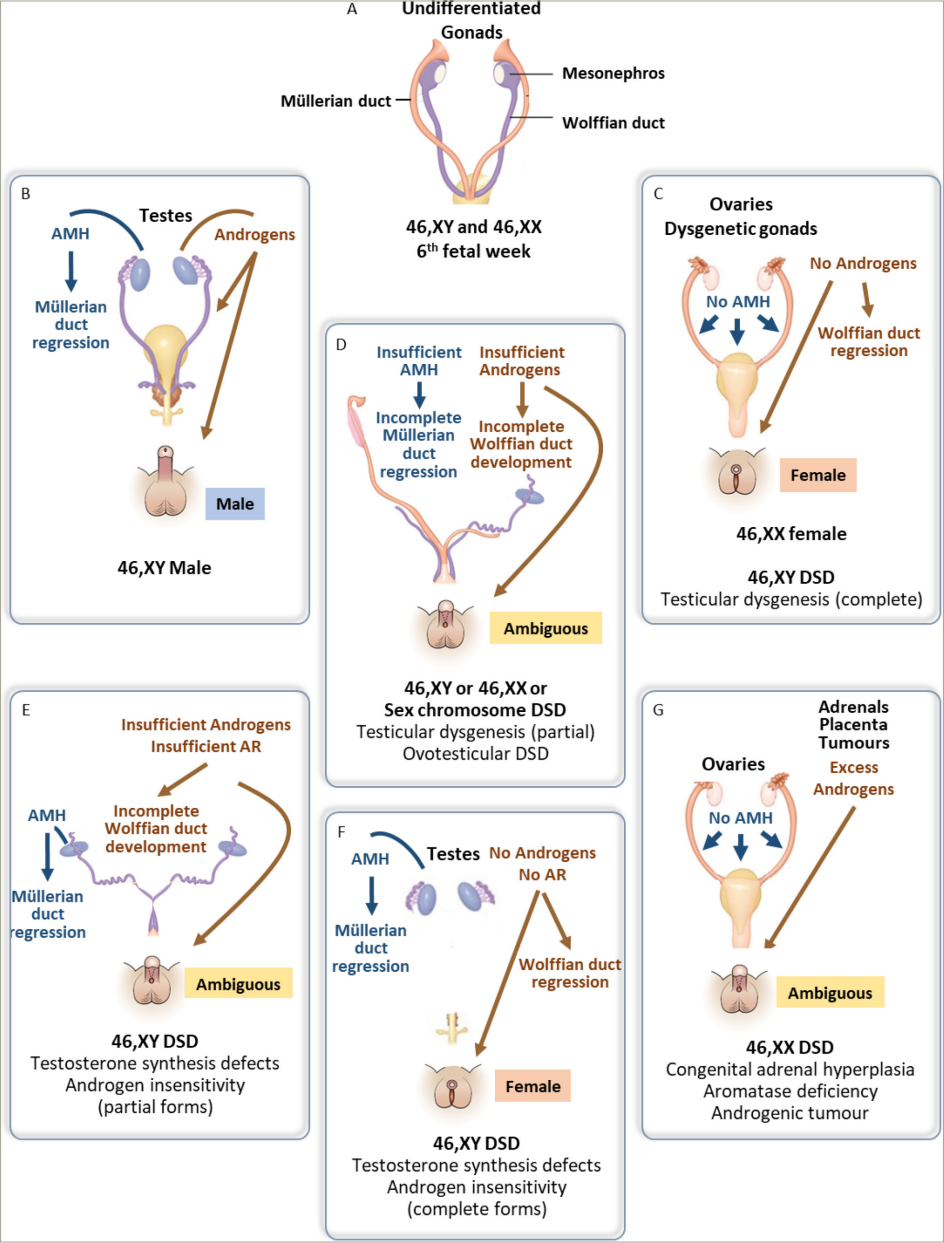
Schematics of gonads and the internal and external genitalia in disorders of sex development^1^

*Schematics of gonads and the internal and external genitalia in (A) the sexually undifferentiated stage of the foetal development and (B–G) 46,XY and 46,XX typical differentiation and the various forms of DSD.*

*AMH = anti-Müllerian hormone; DSD = disorders of sex development.*

*Reproduced and modified with permission, from Josso and Rey.^1^ (http://creativecommons.org/licenses/by-nc/4.0).*

It is now widely accepted that respecting patients’ views is essential, and the most recent guidelines on the management of patients with DSD have included this approach.^11,12^ As stated by the Institute of Medicine Committee on Standards for Developing Trustworthy Clinical Practice Guidelines, “patients and laypersons bring perspectives that clinicians and scientists often lack, and require attention to be paid to those individuals most deeply affected by guidelines.^13^ This input is important not only in deciding what to recommend, but how to present recommendations in ways that are understandable to patients and respectful of their needs”.^13^

On the other hand, the last 20 years have seen healthcare being oriented to “precision” or “personalized” medicine, focusing on individual differences that are not evident phenomenologically; indeed, an important part of the recent research has been driven to identify genes and biomarkers of diseases that help to reach a precise aetiologic diagnosis and predict outcomes.^14,15^ Applied to DSD, this means that we can no longer be satisfied with a diagnosis of 46,XY DSD but that we should attempt to specify whether it is a disorder of gonadal differentiation (i.e. a dysgenetic DSD), and to identify the causing gene variant that could lead, by reverse phenotyping, to the early detection of a clinically inapparent condition in the newborn (e.g. a renal dysfunction or an increased risk for nephroblastoma if gonadal dysgenesis is due to a *WT1* gene variant).^6^ In that sense, a differential characteristic of disciplines with a high degree of consensus is the consistent use of precise terms to refer to each concept.^16^

With the commendable goal of respecting the views and feelings of patients with DSD and their families, many authors have adopted the term “differences of sex development” for DSD and have replaced “ambiguous genitalia” with “atypical genitalia”.^10–12^ In my opinion, the use of less precise terms, such as “differences” and “atypical”, goes against the grain in the era of “precision” medicine. While disorders are differences, not all differences are disorders. “Differences” can be easily understood for gender identity (i.e. a person’s self-representation as female, male or non-binary). Conversely, the conditions under the umbrella term of DSD are specifically disorders (i.e. conditions that lead patients or families to seek healthcare attention). Indeed, although clinical practice has significantly changed in the last 20 years regarding the approach to patients with DSD, a vast majority of these patients will still require healthcare assistance at some point in their lives. For example, hormone replacement will be necessary at the age of puberty and through adulthood in most persons with a diagnosis of DSD, and infertility is a major concern.^11,17^ So far, the best evidence we have to date does not support the perception that the term “disorder of sex development” is insensitive to the concerns of affected persons and that it should therefore be abandoned.^10^

Similarly, the expression “atypical” genitalia has replaced “ambiguous” genitalia. Ambiguous genitalia (i.e. those making the sex assignment difficult) are atypical, but not all atypical genitalia are ambiguous. For instance, penile duplication is a rare form of atypical genitalia, but no sex ambiguity exists.^18^ In addition, this has clinical implications because the diagnostic approach, the aetiologies and the management are completely different. In summary, the medical/scientific community is being contradictory regarding DSD. On the one hand, diagnoses are sought to be more and more precise (e.g. specific pathogenic mechanism involved in a genetic aetiology) in order to apply personalized approaches and treatments. On the other hand, specific (precise) terms, such as “disorders” and “ambiguous”, are being replaced by non-specific (imprecise) ones, such as “differences” and “atypical”. For me, this medical/scientific community's shift may be misleading. For example, guidelines for the diagnosis of patients with DSD recommend performing a number of endocrine tests to reach a precise diagnosis in a newborn with ambiguous genitalia.^10–12^ Those tests are absolutely unnecessary in a newborn with penile duplication or other conditions where genitalia are atypical but not ambiguous.

The intention to “depathologize” DSD is not adequately supported. Instead, I believe we should “dedramatize” the condition. If healthcare professionals, patients’ associations and advocate groups help society to understand that a person with DSD can live a happy life – even if medical attention and treatment are needed – that urinating seated should not be shameful, that a satisfying sex life can be achieved in various ways, etc., the condition will certainly become less dramatic and any term used to describe it will no longer be felt as pejorative or stigmatizing. At present, it is not the terms but the condition itself that stigmatizes. Within the healthcare team and amongst professionals, the most precise terminology should be used to achieve the excellence needed to provide the highest quality of service to their patients while being extremely careful to use adequate, sensitive, not stigmatizing terminology when interacting with patients, their families or society.
